# Intermittent hypoxia increased the expression of ESM1 and ICAM‐1 in vascular endothelial cells via the downregulation of microRNA‐181a1

**DOI:** 10.1111/jcmm.18039

**Published:** 2023-11-15

**Authors:** Shin Takasawa, Mai Makino, Akiyo Yamauchi, Sumiyo Sakuramoto‐Tsuchida, Rina Hirota, Ryusei Fujii, Keito Asai, Yoshinori Takeda, Tomoko Uchiyama, Ryogo Shobatake, Hiroyo Ota

**Affiliations:** ^1^ Department of Biochemistry Nara Medical University Nara Japan; ^2^ Department of Obstetrics and Gynecology Nara Medical University Nara Japan; ^3^ Department of Diagnostic Pathology Nara Medical University Nara Japan; ^4^ Department of Neurology Nara Medical University Nara Japan; ^5^ Department of Respiratory Medicine Nara Medical University Nara Japan

**Keywords:** atherosclerosis, endothelial cell specific molecule‐1, intercellular adhesion molecule‐1, intermittent hypoxia, microRNA‐181a1, sleep apnea syndrome

## Abstract

Sleep apnea syndrome (SAS) exposes cells throughout the body to intermittent hypoxia (IH). Intermittent hypoxia is a risk factor not only for hypertension and insulin resistance but also for vascular dysfunction. We have reported correlations between IH, insulin resistance and hypertension. However, the details of why IH leads to vascular dysfunction remain unclear. In this study, we investigated inflammation‐related transcripts in vascular endothelial cells (human HUEhT‐1 and mouse UV2) exposed to IH by real‐time RT‐PCR and found that *intercellular adhesion molecule‐1* (*ICAM‐1*) and *endothelial cell‐specific molecule‐1* (*ESM1*) mRNAs were significantly increased. ELISA confirmed that, in the UV2 cell medium, ICAM‐1 and ESM1 were significantly increased by IH. However, the promoter activities of *ICAM‐1* and *ESM1* were not upregulated. On the other hand, IH treatment significantly decreased microRNA (miR)‐181a1 in IH‐treated cells. The introduction of miR‐181a1 mimic but not miR‐181a1 mimic NC abolished the IH‐induced upregulation of Ican‐1 and ESM1. These results indicated that ICAM‐1 and ESM1 were upregulated by IH via the IH‐induced downregulation of miR‐181a1 in vascular endothelial cells and suggested that SAS patients developed atherosclerosis via the IH‐induced upregulation of ICAM‐1 and ESM1.

## 1 INTRODUCTION

Sleep apnea syndrome (SAS) is a common sleep disorder characterised by recurrent complete or incomplete pharyngeal obstruction during sleep. It is estimated that, worldwide, nearly 1 billion adults aged 30–69 in the world may suffer from SAS.^1^ It induces apnea and hypopnea, which often result in oxygen desaturation. A growing body of evidence suggests that SAS acts through recurrent episodes of oxygen desaturation and reoxygenation (intermittent hypoxia [IH]), which increases the risk of vascular endothelial dysfunction, hypertension, stroke, heart failure, type 2 diabetes and myocardial infarction.^2^, ^3^, ^4^, ^5^ Sleep apnea syndrome is associated with vascular endothelial dysfunction that is reversible with continuous positive airway pressure.^6^, ^7^, ^8^ Vascular endothelial dysfunction is the earliest predictor of the subsequent development of cardiovascular diseases in the general population.^9^, ^10^, ^11^, ^12^, ^13^, ^14^ Therefore, understanding the mechanism behind vascular endothelial dysfunction in SAS is critical to the understanding and treatment of the cardiovascular consequences of SAS.

Intercellular adhesion molecule‐1 (ICAM‐1) is a cell surface glycoprotein known as an adhesion receptor that directs leukocytes from circulation to sites of inflammation. ICAM‐1 is expressed at low levels in immune cells, endothelial cells and epithelial cells but is known to be upregulated in response to inflammatory stimuli. The function of ICAM‐1 has been best studied in leukocyte trans‐endothelial migration, where ICAM‐1 regulates leukocyte rolling and adhesive interactions with the vessel wall and guides leukocyte crossing of the endothelial layer.^15^ Endothelial cell‐specific molecule 1 (ESM1) is an endothelial cell‐associated proteoglycan and is upregulated by proangiogenic molecules and pro‐inflammatory cytokine stimulation. ESM1 is considered a novel tissue‐ and blood‐based relevant biomarker as it reflects endothelial activation and dysfunction. Recently, serum ESM1 level was revealed to be associated with the severity of SAS and endothelial dysfunction.^16^ MicroRNAs (miRs) are short RNAs of 19 to 25 nucleotides that regulate post‐transcriptional silencing of target gens. A single miR can target hundreds of mRNAs and influence the expression of many genes often involved in a functional interacting pathway.^17^

In the present study, we investigated the direct effects of IH on vascular endothelial cells using human HUEhT‐1 and mouse UV2 vascular endothelial cells and an in vitro IH system. An in vitro IH system is a controlled gas‐delivery system that regulates the flow of nitrogen and oxygen to generate IH.^18^, ^19^ We found that IH significantly increased the mRNA levels of *intercellular adhesion molecule‐1* (*ICAM‐1*) and *endothelial cell‐specific molecule‐1* (*ESM1*) through the downregulation of microRNA (miR)‐181a1.

## 2 MATERIALS AND METHODS

### 2.1 Cell culture

The human vascular endothelial HUEhT‐1 cells, which were established from human umbilical vein endothelial cell line by electroporation of pIRES‐hTERT‐hygr,^20^, ^21^, ^22^ and mouse vascular endothelial UV2 cells^23^, ^24^, ^25^ were purchased from the Japanese Collection of Research Bioresources Cell Bank (Ibaraki, Japan) and RIKEN BioResorce Research Center, respectively. The HUEhT‐1 cells were grown in MCDB131 medium (Gibco) containing 10% (v/v) foetal calf serum (FCS), 10 mM L‐glutamine, 5 μg/mL heparin, 30 mg/L endothelial cell growth supplement (Corning, Corning, NY), 100 units/mL penicillin G (FUJIFILM Wako) and 100 μg/mL streptomycin (FUJIFILM Wako). The mouse vascular endothelial UV2 cells were grown in DMEM medium (FUJIFILM Wako) containing 10% (v/v) FCS, 100 units/mL penicillin G (FUJIFILM Wako) and 100 μg/mL streptomycin (FUJIFILM Wako). The cells were exposed to either normoxia (21% O_2_, 5% CO_2_ and balanced N_2_) or IH (70 cycles of 5 min sustained hypoxia (1% O_2_, 5% CO_2_ and balanced N_2_) and 10 min normoxia) in a custom‐designed, computer‐controlled incubation chamber attached to an external O_2_‐CO_2_‐N_2_ computer‐driven controller (O_2_ programmable control, 9200EX, Wakenbtech), as described in previous works.^26^, ^27^, ^28^, ^29^, ^30^, ^31^, ^32^ We used this in vitro model of IH, which resulted in fluctuations in the pressure of oxygen similar to the IH condition observed in patients with severe SAS, to repeatedly expose the cells to severe hypoxemia followed by mild hypoxemia or normoxia (i.e. IH).^33^ We have previously reported that the magnitude of the IH expressed by SpO_2_ fluctuated between 75% and 98% and between 50% and 80% in patients with SAS,^18^, ^19^ which was nearly equivalent to the medium condition in the present study.

### 2.2 Real‐time RT‐PCR

Total RNA was isolated from HUEhT‐1 and UV2 cells using an RNeasy Plus Mini Kit (Qiagen), and cDNA was synthesised from total RNA as a template using a High Capacity cDNA Reverse Transcription kit (Applied Biosystems, Foster City, CA), as previously described.^26^, ^27^, ^28^, ^29^, ^30^, ^31^, ^32^, ^34^, ^35^ Total RNA, including miRNA, was isolated from UV2 cells using the miRNeasy mini kit (Qiagen) according to the manufacturer's instructions. An equal amount of DNase‐treated RNA was poly‐A‐tailed using a Mir‐X™ miRNA first‐strand synthesis kit (Clontech Laboratories, Inc., Mountain View, CA) according to the manufacturer's protocol. A real‐time PCR was performed using an SYBR® Fast qPCR kit (KAPA Biosystems) and a Thermal Cycler Dice Real Time System (Takara Bio). All the PCR primers were synthesised by Nihon Gene Research Laboratories, Inc. (NGRL) as follows: 5′‐TTGCTGACAGCTGACCTTTG‐3′ and 5′‐TTTAGGCCACATTGGGAAAG‐3′ for human vascular cell adhesion molecule‐1 (*VCAM‐1*) (NM_001078.4), 5′‐CCTGCCCCAATCCCTTTATT‐3′ and 5′‐CCCTAAGCCCCCAATTCTCT‐3′ for human *tumour necrosis factor‐α* (*TNFα*) (NM_000594.4), 5′‐GGCTGGAGCTGTTTGAGAAC‐3′ and 5′‐ACTGTGGGGTTCAACCTCTG‐3′ for human *ICAM‐1* (NM_000201.3), 5′‐AAGAAACCACCGGAAGGAAC‐3′ and 5′‐ACTCCTTGGCAAAACTGCAC‐3′ for human *interleukin‐8* (*IL‐8*) (NM_000584.4), 5′‐CACAGACTTAGTGGCCATCCA‐3′ and 5′‐TCTTTCGGATCCCAATCCA‐3′ for human *P‐selectin* (*P‐SEL*) (NM_003005.4), 5′‐CGTGCCCACATCAAGGAG‐3′ and 5′‐GGACAAGAGCAAGCAGAAAC‐3′ for human *C‐C motif chemokine ligand 5* (*CCL5*) (NM_002985.3), 5′‐TTCCATTTGCAAGGGAAAAG‐3′ and 5′‐ACACACAGCCAGTCAACGAG‐3′ for human C‐X‐C motif chemokine ligand 12 (*CXCL12*) (NM_000609.7), 5’‐CAGGCATGGATGGCATGAAG‐3′ and 5’‐CTGACTGGCAGTTGCAGGTCTC‐3′ for human *ESM1* (NM_007036.5), 5′‐CTCACCGCTACAACATCCTG‐3′ and 5′‐TTTCCACAGGGACGAGGT‐3′ for human nitric oxide synthase 3 (*NOS3*) (NM_000603.5), 5′‐*GAGAAACCCACTCCCAGTCC*‐3′ and 5′‐*GATGTCCAGGTGGCAGAAGT*‐3′ for human endothelin‐1 (*EDN‐1*) (NM_001955.5), 5´‐GCGAGAAGATGACCCAGA‐3′ and 5´‐CAGAGGCGTACAGGGATA‐3′ for human *β‐actin* (NM_001101), 5′‐CCCCAAGGATCCAGAGATTCA‐3′ and 5’‐ACTTGACCGTGACCGGCTT‐3′ for mouse *Vcam‐1* (NM_011693.3), 5’‐CATCTTCTCAAAATTCGAGTGACAA‐3′ and 5’‐TGGGAGTAGACAAGGTACAACCC‐3′ for mouse *Tnfα* (NM_013693.3), 5’‐ATCTCAGGCCGCAAGGG‐3′ and 5′‐CGAAAGTCCGGAGGCTCC‐3′ for mouse *ICAM‐1* (NM_010493.3), 5′‐CGGCAATGAAGCTTCTGTAT‐3′ and 5′‐CCTTGAAACTCTTTGCCTCA‐3′ for mouse *Il‐8* (NM_011339.2), 5′‐GGGCTCAACTCATCTGGTTC‐3′ and 5′‐CATTGAGGTGAGCGATTTCA‐3′ for mouse *P‐sel* (NM_011347.2), 5′‐GGTACCATGAAGATCTCTGC‐3′ and 5′‐CTATCCTAGCTCATCTCC‐3′ for mouse *Ccl5* (NM_013653.3), 5′‐GGAGGATAGATGTGCTCTGGAAC‐3′ and 5′‐AGTGAGGATGGAGACCGTGGTG‐3′ for mouse *Cxcl12* (NM_001012477.2), 5′‐GGAGGATGATTTTGGTGACG‐3′ and 5′‐CTGTCACATATGCCCGACTG‐3′ for mouse *ESM1* (NM_023612.3), 5′‐AGCATACCCCCACTTCTGTG‐3′ and 5′‐GAAGATATCTCGGGCAGCAG‐3′ for mouse *Nos3* (NM_008713.4), 5′‐CCTGGACATCATCTGGGTC‐3′ and 5′‐TGTGGCCTTATTGGGAAG‐3′ for mouse *End‐1* (NM_010104.4), 5′‐CTCTTTCCCACCCAGTGCTA‐3′ and 5′‐TGGTCGTCGTAGTGCTTGAG‐3′ for mouse *Drosha* (NM_001130149.1), 5′‐ATGCAAAAAGGACCGTGTTC‐3′ and 5′‐CAAGGCGACATAGCAAGTCA‐3′ for mouse *Dicer* (NM_148948.2), 5′‐GAACATTCAACGCTGTCGGTG‐3′ and 5′‐GGTCGATGGTTTTTATTTGAATTCC‐3′ for mouse *miR‐181a1* (NR_029795.1), 5′‐GAACATTCAACGCTGTCGGTG‐3′ and 5′‐GGTACAGTCAACGGTCGGTGG‐3′ for mouse *miR‐181a2* (NR_029568.1), 5′‐TCAACATTCATTGCTGTCGGTG‐3′ and 5′‐GTTGCATTCATTGTTCAGTGAGC‐3′ for mouse *miR‐181b1* (NR_029820.1), 5′‐TCAACATTCATTGCTGTCGGTG‐3′ and 5′‐CGCAGTTTGCATTCATTGATCAG‐3′ for mouse *miR‐181b2* (NR_029904.1), 5′‐GGGAACATTCAACCTGTCGG‐3′ and 5′‐GGTCCACTCAACGGTCGATGG‐3′ for mouse *miR‐181c* (NR_029821.1), 5′‐TTAACATTCATTGTTGTCGGTGG‐3′ and 5′‐TGGGTCTGGCTGCCTCCTCAC‐3′ for mouse *miR‐181d* (NR_030534.1), 5´‐ACGGCAAGACCTTCAACCAG‐3′ and 5´‐ATGGAGAACTCGCCCAGGTAG‐3′ for mouse *rat insulinoma gene*/*ribosomal protein S15* (*Rig*/*RpS15*) (NM_009091.2) and 5′‐CGCTTCGGCAGCACATATAC‐3′ and 5′‐AAATATGGAACGCTTCACGA‐3′ for mouse U6 (XR_004940589.1). The PCR was performed using an initial step of 3 min at 95°C followed by 40 cycles of 3 s at 95°C and 20 s at 60°C for *β‐actin*; 45 cycles of 10 s at 95°C, 5 s at 60°C and 20 s at 72°C for *Rig*/*RpS15*; and 45 cycles of 3 s at 95°C and 20 s at 60°C for *Vcam‐1* (human and mouse), *Tnfα* (human and mouse), *ICAM‐1* (human and mouse), *Il‐8* (human and mouse), *P‐sel* (human and mouse), *Ccl5* (human and mouse), *Cxcl12* (human and mouse), *ESM1* (human and mouse), *Nos3* (human and mouse), *End‐1* (human and mouse), mouse *Drosha* and mouse *Dicer* and mouse *microRNA‐181a1* (*miR‐181a1*), *miR‐181a2*, *miR‐181b1*, *miR‐181b2*, *miR‐181c* and *miR‐181d*. The mRNA expression levels were normalised to the mRNA level of *Rig*/*RpS15* in mouse samples or *β‐actin* in human samples, and the mouse miR levels were normalised to the *U6* RNA level.

### 2.3 Measurements of ICAM‐1 and ESM1 in the culture medium by enzyme‐linked immunosorbent assay (ELISA)

The mouse UV2 vascular epithelial cells were exposed to either normoxia or IH for 24 h. Then, the culture medium was collected, and the ICAM‐1 and ESM1 concentrations were determined by using a Mouse ICAM‐1/CD54 Sandwich ELISA Kit (Proteintech) and Mouse ESM1 ELISA Kit (Wuhan Fine Biotech Co., Ltd.) according to the supplier's instructions.

### 2.4 Construction of reporter plasmid and luciferase assay

Reporter plasmids were prepared by inserting the promoter fragments of human *ICAM‐1* (−1792 ~ +43: AY225514) and *ESM1* (−1180 ~ +37: NC_000005) upstream of a luciferase reporter gene in the pGL4.17 vector (Promega). As the transfection efficiency of HUEhT‐1 human vascular endothelial cells was very much lower, the reporter plasmids were transfected into mouse UV2 vascular endothelial cells using Lipofectamine® 3000 (Invitrogen), as previously described,^28^, ^29^, ^30^, ^31^, ^32^, ^35^ and the cells were exposed to either 70 cycles/24 h of IH or normoxia for 24 h. After the cells were exposed to IH, they were harvested, and cell extracts were prepared in Extraction Buffer (0.1 M potassium phosphate, pH 7.8/0.2% Triton X‐100; Life Technologies). The pCMV‐SPORT‐βgal plasmid (Life Technologies) was co‐transfected in all experiments at a 1:10 dilution for monitoring transfection efficacy. Luciferase activity was measured using a PicaGene luciferase assay system (Toyo‐ink) and was normalised by using β‐galactosidase activity, as described previously.^26^, ^28^, ^29^, ^30^, ^31^, ^32^, ^35^

### 2.5 RNA interference

The small interfering RNA (siRNAs) against the mouse *ESM1* and *ICAM‐1* were synthesised by NGRL. The sense sequence of the siRNAs for the mouse *ESM1* and *ICAM‐1* were 5′‐UUCUCCAGCACCCAUAUGUtt‐3′ (corresponding to the 1512–1530 of NM_023612.3) and 5′‐CCAACUGGAAGCUGUUUGAtt‐3′ (corresponding to the 313–331 of NM_010493.3), respectively. The Silencer® Select scrambled siRNA Ambion was used as a control. The transfection of the siRNA into the UV2 vascular endothelial cells was carried out using the Lipofectamine® RNAiMAX Transfection Reagent (Invitrogen). The cells were each transfected with 5 pmol of siRNA in a 24‐well culture dish, as described in prior works.^27^, ^28^, ^29^, ^31^, ^32^

### 2.6 MiR‐181a1 mimic transfection

MiR‐181a1 mimic (5′‐AACAUUCAACGCUGUCGGUGAGUtt‐3′, 5′‐ACUCACCGACAGCGUUGAAUGUUtt‐3′: corresponding to 14–36 of NR_029795.1) and non‐specific control RNA (miR‐181a1 mimic NC) (5′‐UUUGUACUACACAAAAGUACUGtt‐3′, 5′‐CAGUACUUUUGUGUAGUACAAAtt‐3′) were synthesized via NGRL and introduced into UV2 cells using Lipofectamine® RNAiMAX (Invitrogen)^28^, ^29^, ^30^, ^35^ just prior to IH/normoxia exposure, and the mRNA levels of *ESM1* and *ICAM‐1* were measured via real‐time RT‐PCR, as previously described in 2.2.

### 2.7 Data analysis

The results are expressed as mean ± SE. Statistical significance was determined via Student's *t*‐test using GraphPad Prism version 8.4.3 (GraphPad Software).

## 3 RESULTS

### 3.1 The gene expression of ESM1 and ICAM‐1 in vascular endothelial cells were increased by IH

**FIGURE 1:**
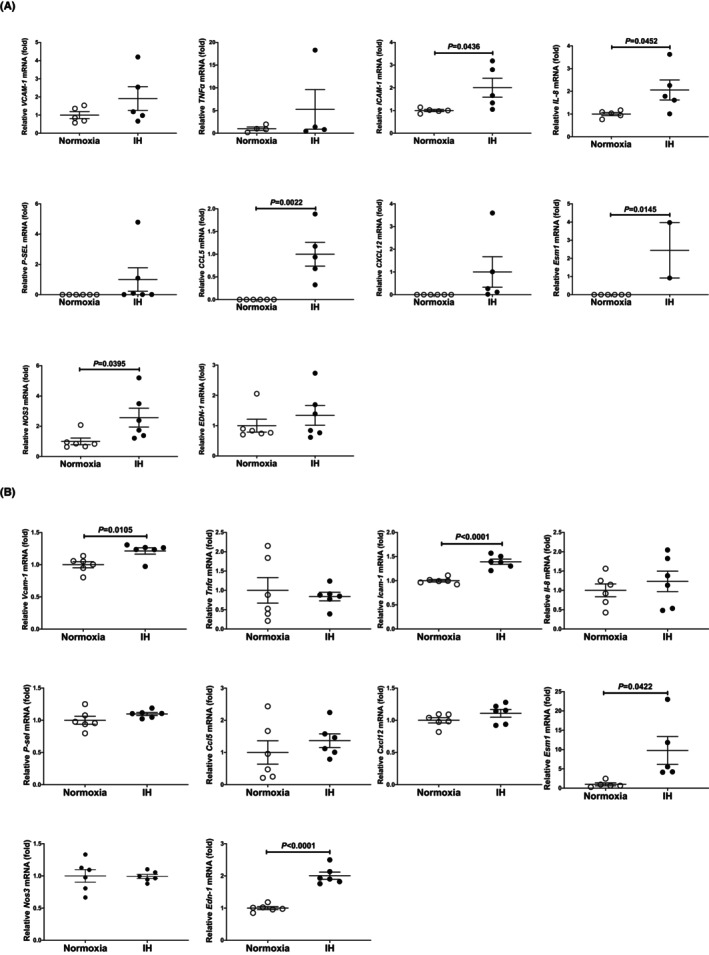
The mRNA levels of vascular endothelial inflammatory transcripts in (A) human vascular HUEhT‐1 endothelial cells and (B) mouse vascular UV2 endothelial cells. Human HUEhT‐1 and mouse UV2 vascular endothelial cells were treated with normoxia or IH for 24 h. The mRNA levels were measured via real‐time RT‐PCR and normalised using *β‐Actin* in human HUEhT‐1 cells and *rat insulinoma gene* (*Rig*)/*ribosomal protein S15* (*RpS15*) in mouse UV2 cells as an internal standard. In HUEhT‐1. The mRNAs of *P‐SEL*, *CCL5*, *CXCL12* and *ESM1* in normoxia were not detected in HUEhT‐1 cells. Therefore, the mRNA levels exposed to normoxia were set to 1.0 except for *P‐SEL*, *CCL5*, *CXCL12* and *ESM1*. Data are expressed as the mean ± SE of the samples. Statistical analyses were performed using Student's *t*‐test. Intermittent hypoxia significantly increased the mRNA levels of *ICAM‐1*, *IL‐8*, *CCL5*, *ESM1* and *NOS3* in human HUEhT‐1 cells. The mRNA expressions of *VCAM‐1*, *TNFα*, *P‐SEL*, *CXCL12* and *EDN‐1* were not significantly increased by IH (*p* = 0.2172, *p* = 0.3696, *p* = 0.2770, *p* = 0.1336 and *p* = 0.4022, respectively). Intermittent hypoxia significantly increased the mRNA levels of *Vcam‐1*, *ICAM‐1*, *ESM1* and *Edn‐1* in mouse UV2 cells.

Human vascular endothelial HUEhT‐1 cells and mouse vascular endothelial UV2 cells were exposed to normoxia or IH for 24 h. Following the IH/normoxia exposure, cellular RNA was prepared, and the mRNA levels of endothelial inflammatory transcripts were measured. Several mRNAs encoding *ICAM‐1*, *interleukin IL‐8*, *CCL5*, *ESM1* and *NOS3* were upregulated in response to IH in human HUEhT‐1 vascular endothelial cells (Figure 1A), and the mRNAs of *Vcam‐1*, *ICAM‐1*, *ESM1* and *Edn‐1* were upregulated by IH in mouse UV2 endothelial cells (Figure 1B). *ICAM‐1* and *ESM1* were commonly upregulated by IH in human HUEhT‐1 and mouse UV2 vascular endothelial cells.

**FIGURE 2:**
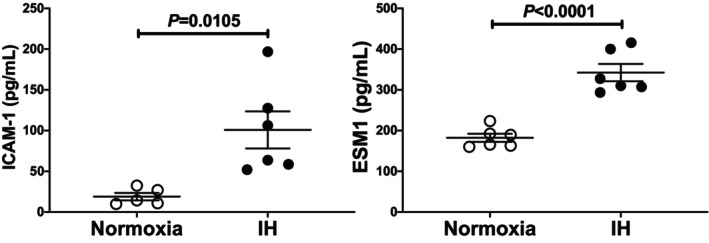
Concentrations of ICAM‐1 and ESM1 in UV2 mouse vascular endothelial cell culture medium. Mouse UV2 vascular endothelial cells were treated with normoxia or IH for 24 h. The ICAM‐1 (left panel) and ESM1 (right panel) concentrations in the medium were measured via ELISA. Data are expressed as mean ± SE for each group. Statistical analyses were performed using Student's *t*‐test.

We further measured the protein levels of ICAM‐1 and ESM1 in mouse UV2 cell culture medium by ELISA and found that the ICAM‐1 and ESM1 levels were significantly increased by IH: ICAM‐1 (*p* = 0.0105) and ESM1 (*p* < 0.0001) (Figure 2).

### 3.2 The promoter activities of *ICAM‐1* and *ESM1* were not increased by IH

**FIGURE 3:**
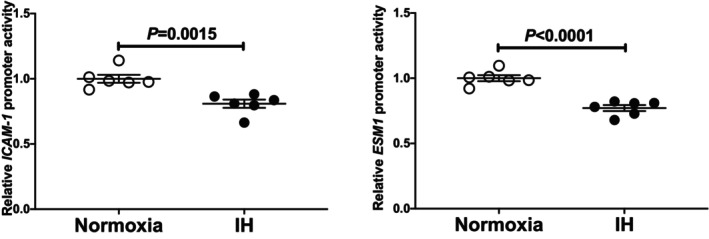
Promoter activities of *ICAM‐1* (left panel) and *ESM1* (right panel) in mouse UV2 vascular endothelial cells. Reporter plasmids prepared by inserting the promoter fragments of human *ICAM‐1* (−1792 ~ +43) and *ESM1* (−1180 ~ +37) upstream of a firefly luciferase reporter gene in a pGL4.17 vectors were transfected into the UV2 cells. After the cells were exposed to either IH or normoxia for 24 h, they were lysed, and the promoter activities of *ICAM‐1* and *ESM1* were measured. All data are represented as the mean ± SE of the samples of six independent experiments. The promoter activities exposed to normoxia were set to 1.0. The statistical analyses were performed using Student's *t*‐test.

In order to determine whether the IH‐induced increases in *ICAM‐1* and *ESM1* mRNAs were caused by their transcription, the human *ICAM‐1* promoter (−1795 ~ +43) and the human *ESM1* promoter (−1180 ~ +37) were fused to the luciferase gene of pGL4.17 and transfected them into mouse UV2 vascular endothelial cells. After IH stimulation, we measured promoter activities and found that *ICAM‐1* and *ESM1* promoter activities were not increased but, rather, decreased by IH in UV2 cells (Figure 3: *p* = 0.0015 in *ICAM‐1* promoter and *p* < 0.0001 in *ESM1* promoter). These results suggested that the gene expression of *ICAM‐1* and *ESM1*, overexpressed by IH, was not regulated by transcription.

### 3.3 Downregulation of ESM1 by siRNA for ESM1 attenuated the ICAM‐1 increase in UV2 cells treated with IH

**FIGURE 4:**
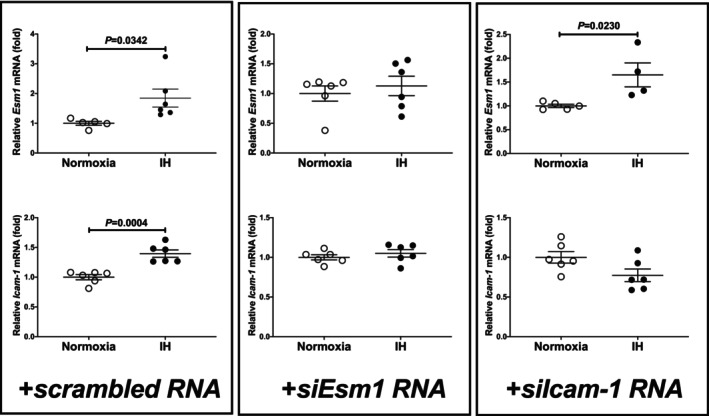
Effects of the siRNAs against *ESM1 and ICAM‐1* on the IH‐induced gene expression of *ICAM‐1* and *ESM1*. The siRNAs for *ESM1* and *ICAM‐1* were transfected into the UV2 vascular endothelial cells, and the cells were then subjected to IH or normoxia for 24 h. The levels of the *ESM1* and *ICAM‐1* mRNAs were measured via a real‐time RT‐PCR using *Rig*/*RpS15* as an endogenous control. The mRNA levels exposed to normoxia were set to 1.0. The data are expressed as the mean ± SE for each group of six independent experiments (*n* = 6). The statistical analyses were performed using Student's *t*‐test.

To see the mechanism of expressions of *ICAM‐1* and *ESM1* in the UV2 cells, the *ICAM‐1* and *ESM1* genes were knocked down using siRNA. The expression of *ICAM‐1* and *ESM1* was significantly increased by IH in the presence of scrambled RNA (Figure 4, left panel). In contrast, the introduction of the siRNA for ESM1 (*siESM1*) inhibited not only the IH‐induced increases in the mRNAs for *ESM1* but also the IH‐induced increase in *ICAM‐1* levels in the UV2 cells (Figure 4, middle panel). However, the introduction of siRNA for ICAM‐1 (*siIcam1*) clearly inhibited the IH‐induced increase in *ICAM‐1* mRNA but unchanged the IH‐induced increase in *ESM1* mRNA (Figure 4 right panel). These results strongly suggested that the increases in the *ICAM‐1* by IH (Figure 1A,B) were caused by the increased *ESM1* expression.

### 3.4 The miR‐181a1 level was significantly decreased by IH

**FIGURE 5:**
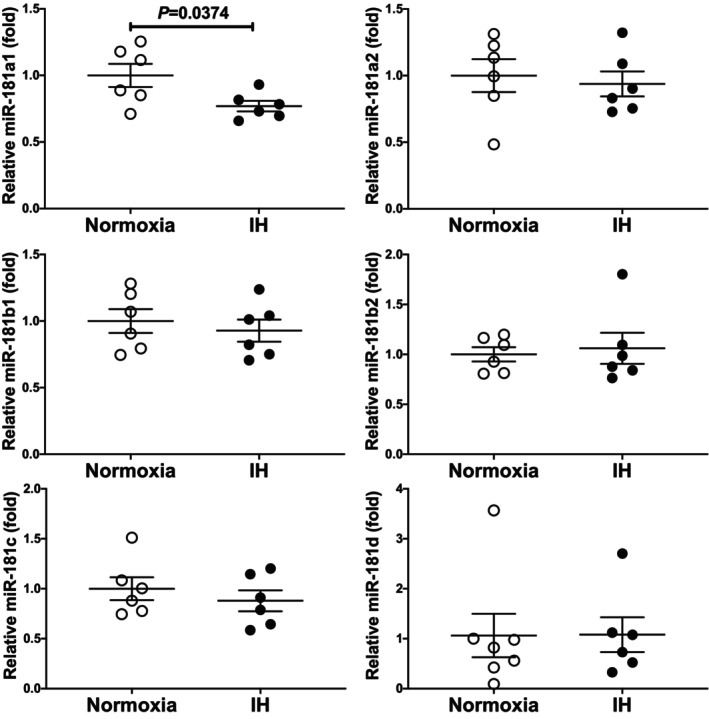
Expression of miR‐181 family in UV2 vascular endothelial cells. Mouse UV2 vascular endothelial cells were treated with normoxia or IH for 24 h. The miR levels were measures by real‐time RT‐PCR and normalised using U6 RNA as an internal standard. The miR levels exposed to normoxia were set to 1.0. Data are expressed as the mean ± SE of the samples. Statistical analyses were performed using Student's *t*‐test. Intermittent hypoxia significantly decreased the miR‐181a1 level in mouse UV2 cells.

**FIGURE 6:**
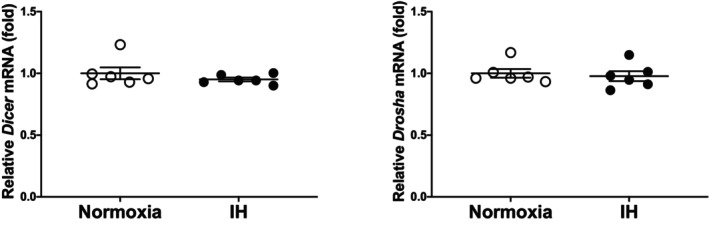
The levels of the Dicer mRNA (left panel) and Drosha mRNA (right panel) of the UV2 mouse vascular endothelial cells subjected to normoxia or IH for 24 h. The levels of the Dicer and Drosha mRNAs were measured by means of a real‐time RT‐PCR using Rig/RpS15 as an endogenous control. The data are expressed as the mean ± SE for each group of six independent experiments. The mRNA levels of cells exposed to normoxia were set to 1.0. The statistical analyses were performed using Student's *t*‐test.

We considered a potential explanation for the IH‐induced upregulation of *ICAM‐1*, and *ESM1* was under the post‐transcriptional control via *ESM1* expression. Therefore, we searched for targeted miRNA using the MicroRNA.org programme (http://www.microrna.org/microrna/home.do) and found that human *ESM1* and mouse *ESM1* mRNAs have a potential target sequence for miR‐181 family. There was no other miRNA candidate targeting for both genes. We measured all the miR‐181 family in IH‐treated UV2 cells by real‐time RT‐PCR and found that the miR‐181a1 level was significantly lower than that of normoxia‐treated cells (Figure 5: 0.7694 fold vs. normoxia, *p* = 0.0374), but the other members of miR‐181 family were unchanged (0.9377 fold in miR‐181a2, 0.9282 fold in miR‐181b1, 1.060 fold in miR‐181b2, 0.8790 fold in miR‐181c and 1.080 fold in miR‐181d; *p* = 0.6960, 0.5696, 0.7232, 0.4543 and 0.9767, respectively). There could be several reasons as to why miR‐181a1 was specifically decreased among miR‐181 family by IH. One is that the enzymes involved in miRNA biogenesis are decreased by IH, and as a result the level of miR‐181a was decreased. Another is that the level of miR‐181a1 was specifically decreased by IH. We measured the mRNAs of *endoribonuclease Dicer* (*Dicer*) and *ribonuclease type III* (*Drosha*), which are involved in the biosynthesis of miRNAs^36^, ^37^ and found that their expression levels were unchanged by IH (Figure 6: *p* = 0.3501 in *Dicer* and *p* = 0.6784 in *Drosha*). These results suggest that the reduction of miR‐181a1 plays a key role in the post‐transcriptional upregulation of mRNA levels of *ESM1*, leading to the upregulation of *ICAM‐1* mRNA. In order to clarify whether *ESM1* and *ICAM‐1* expression in IH is regulated by miR‐181a1, miR‐181a1 mimic and miR‐181a1 mimic NC (non‐specific control RNA) were introduced into UV2 vascular endothelial cells, and cells were exposed to IH or normoxia. We prepared RNA from the cells, and the mRNA levels of *ESM1* and *ICAM‐1* were measured by RT‐PCR.

**FIGURE 7:**
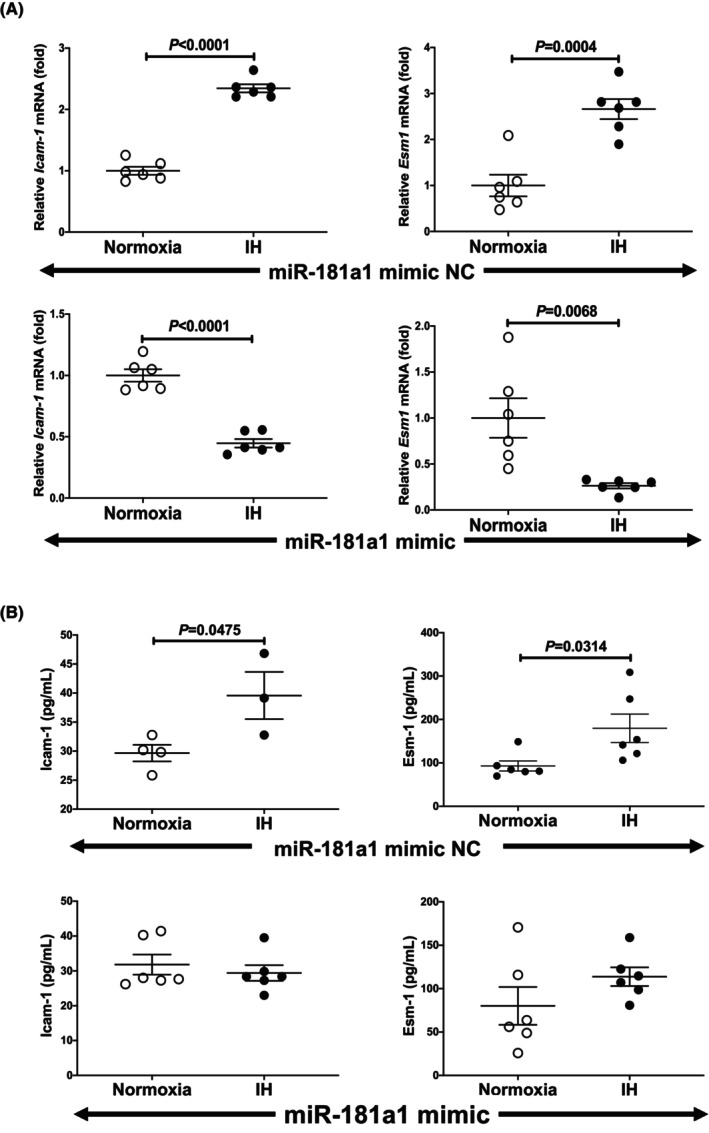
Effects of miR‐181a1 mimic transfection on the ICAM‐1 and ESM1 expression. The miR‐181a1 mimic (5´‐AACAUUCAACGCUGUCGGUGAGUtt‐3′, 5´‐ACUCACCGACAGCGUUGAAUGUUtt‐3′) and the non‐specific control RNA (miR‐181a1 mimic NC) (5´‐UUUGUACUACACAAAAGUACUGtt‐3′, 5´‐CAGUACUUUUGUGUAGUACAAAtt‐3′) were synthesized by Nihon Gene Research Laboratories, Inc. (NGRL). They were introduced into the UV2 vascular endothelial cells using Lipofectamine® RNAiMAX just prior to the IH/normoxia exposure. (A) The mRNA levels of ICAM‐1 and ESM1 were measured by means of a real‐time RT‐PCR, as described in the Materials and Methods section, using *Rig*/*RpS15* as an endogenous control. The data are expressed as the mean ± SE for each group of six independent experiments. The mRNA levels of cells exposed to normoxia were set to 1.0. The statistical analyses were performed using Student's *t*‐test. (B) The protein levels of ICAM‐1 and ESM1 were measured using ELISA, as described in the Materials and Methods section. The data are expressed as the mean ± SE for each group of six independent experiments. In normoxia‐ and IH‐treated cells, in the cell medium to which miR‐181a1 mimic was introduced cell, ICAM‐1 and ESM1 were unchanged (*p* = 0.5226 and *p* = 0.1959, respectively). The statistical analyses were performed using Student's *t*‐test.

As shown in Figure 7A, we found that the IH‐induced increases in the *ICAM‐1* and *ESM1* mRNAs were abolished by the introduction of the miR‐181a1 mimic but not by miR‐181a1 mimic NC. The increases of ICAM‐1 and ESM1 in the medium protein in response to IH were also abolished by the introduction of the miR‐181a1 mimic (Figure 7B). These results indicated that IH stress downregulates the miR‐181a1 in UV2 vascular endothelial cells, resulting the levels of the *ESM1* mRNA are increased via the miR‐181a1‐mediated mechanism.

## 4 DISCUSSION

Sleep apnea syndrome patients as well as chronic obstructive lung diseases, smoking, sickle cell anaemia and/or immaturity of newborns,^38^, ^39^ and their organs, tissues and cells are exposed to IH during sleep, and in SAS patients, several complications, such as diabetes, hypertension and cardiovascular dysfunction are caused by IH‐induced abnormal gene expression.^18^, ^19^, ^26^, ^27^, ^28^, ^29^, ^30^, ^31^, ^32^ Intermittent hypoxia caused by SAS can lead to systemic tissue hypoxia and many other maladaptive effects, including oxidative stress, mitochondrial dysfunction, inflammation and hyperactivation of the sympathetic nervous system. Vascular fibrosis, characterised by reduced lumen diameter and thickened arterial wall resulting from excessive deposition of extracellular matrix, is associated with many clinical diseases and pathological progressions, including atherosclerosis. Although vascular endothelial dysfunction, the earliest predictor of the subsequent development of cardiovascular diseases, has been reported in SAS patients, the mechanism explaining why the endothelial complications were induced by IH remained unclear. In the present study, we exposed human and mouse vascular endothelial cells to IH, analysed gene expression, and found that IH exposure induced increases in *ICAM‐1* and *ESM1* mRNAs. We further examined the mechanisms how IH upregulates the mRNA levels of both *ICAM‐1* and *ESM1* and identified potential post‐transcriptional miR‐181a1‐mediated regulated mechanisms.

Recent epidemiological research has demonstrated that SAS may be associated with various metabolic dysfunctions, including dyslipidemia, cardiovascular diseases, insulin resistance and hypertension, and the dysfunctions may lead vascular dysfunction. The pathophysiology of vascular endothelial dysfunction in relation to SAS is dependent on various factors, for example, oxidative stress, lipid peroxidation, protein oxidation, DNA oxidation, nitric oxide‐related compounds, and inflammatory markers.^40^ Cell adhesion molecules are a subset of cell adhesion proteins located on the cell surface and play an important role in enabling cells to bind to other cells or with extracellular matrix molecules. The immunoglobulin superfamily is one of the largest superfamilies of proteins in the body, and it includes many diverse cell adhesion molecules, such as Icam(s) and Vcam(s), which undergo heterophilic binding with molecules such as carbohydrates and integrins.^41^ Interestingly, both Icam(s) and Vcam(s) are endothelial cell adhesion molecules of the immunoglobulin superfamily with a crucial role in mediating the firm adhesion of leukocytes to endothelial cells that are expressed on vascular endothelial cells and interact with integrins such as CD11/18 on leukocytes to assist with leukocyte adhesion and trafficking. Five members of the Icam immunoglobulin superfamily have been identified: ICAM‐1 to −5. ICAM‐1 is a major adhesion receptor for monocyte/macrophage attachment to vascular endothelial cells at inflammatory sites through binding to its receptors leukocyte function‐associated antigen 1 and macrophage‐1 antigen.^42^, ^43^, ^44^ Moreover, ICAM‐1 expression is induced by various inflammatory stimuli.^43^ An TNFα treatment markedly upregulates ICAM‐1 expression on the surface of endothelial cells.^45^ Pressure overload induces ICAM‐1 expression in the endothelial cells of myocardial arterioles.^43^

Predictors of vascular risk, including obesity, hyperlipidemia, metabolic syndrome, diabetes mellitus and hypertension, are indeed associated with the presence of vascular endothelial dysfunction. ESM1, also called endocan, is a novel inflammatory marker,^46^ and elevated levels have been reported in some endothelial dysfunction‐related diseases.^47^ ESM1 is a soluble proteoglycan of 50 kDa that is secreted by the vascular endothelium and can be detected in the blood.^48^ ESM1 is involved in molecular interactions with a wide range of biologically active moieties, which are essential for the regulation of biological processes such as cell adhesion, migration and proliferation. ESM1 also plays a role in endothelium‐dependent pathological disorders and may be a surrogate endothelial dysfunction marker.^49^ Clinical data indicate that ESM1 is positively correlated with hypertension. For every 1 pg/mL increase in ESM1, the incidence of hypertension increased by 32.2%.^50^ In the early stages of hypertension, the ESM1 concentration in plasma is significantly increased. ESM1 levels are positively correlated with renal enzymes, norepinephrine,^51^ carotid intima‐media thickness and high‐sensitivity C‐reactive protein, as well as being negatively correlated with leukocyte count.^51^ There is a correlation between the ESM1 level and the occurrence of heart failure.^52^ ESM1 is an independent predictor of heart failure‐related events in chronic heart disease patients. Serum ESM1 levels in patients with aortic atherosclerosis strokes are significantly increased. Higher ESM1 levels in the serum of patients with aortic atherosclerotic stroke can help predict short‐term adverse outcomes.^53^

We investigated the mechanisms by which IH upregulates the mRNA levels of *ICAM‐1* and *ESM1* and found that the promoter activities of the genes were not increased by IH, suggesting that the IH‐induced upregulation of the *ICAM‐1* and *ESM1* is regulated during the post‐transcriptional step. MiRNAs are small non‐coding RNAs (~22 nucleotides in length) that modulate gene expression via either translational suppression or the degradation of the mRNA through binding to the 3′‐untranslated regions of the target genes in a base‐pairing manner.^54^ They affect the stability of the target mRNAs, resulting in changes in the target mRNA, which is one of the mechanisms associated with post‐transcriptional regulation. To date, a number of studies concerning the role of the miR‐181 family (miR‐181a1, −181a2, −181b1, −181b2, −181c and −181d) in the development, differentiation, neurodegenerative diseases and cancer.^55^ Several studies addressed the correlation between miRNAs and vascular endothelial dysfunction in SAS patients. For example, circulating exosomal miR‐630 was reported to be a key mediator of vascular dysfunction,^56^ and circulating extracellular vesicles containing miR‐144 have been found to regulate endothelial function, reducing nuclear factor erythroid 2‐related factor 2 expression.^57^ Concerning the roles of miR‐181a1, Cheng et al. reported that miR‐181a1 was a novel inhibitor of mitophagy.^58^ Cao et al. reported that miR‐181 impeded IL‐17‐induced non‐small cell lung cancer proliferation and migration through targeting VCAM‐1 expression.^59^ Lin et al.^60^ reported that miR‐181 functioned as an anti‐oncogene via NFκB pathway by targeting Rhotekin2 in ovarian cancers. In the present study, the decline of the miR‐181a1, with a target sequence in the *ESM1* mRNA, could have contributed to the worsening of vascular dysfunction in the IH‐condition, inducing the upregulation of the ESM1 and ICAM‐1 mRNAs. We initially have some other possibility to induce vascular endothelial dysfunction by IH such as RhoA/Rho kinase‐mediated mechanism.^61^ However, *NOS3* was upregulated in human HUEhT‐1 cells but not upregulated in mouse UV2 cells. In contrast, *Edn‐1* upregulation by IH was observed in mouse UV2 cells but not in human HUEhT‐1 cells (Figure 1A,B).

In summary, the gene expression of *ESM1* and *ICAM‐1* were increased via the downregulation of the miR‐181a1 in the IH‐treated vascular endothelial cells, suggesting that, in SAS patients, the upregulation of *ESM1* and *ICAM‐1* may induce vascular endothelial dysfunction, while miR‐181a1 could play a crucial role in the regulation of such gene expressions. Analyses of miR‐181a1 in animal model and/or SAS patient samples could be next step to reveal the mechanisms.

## AUTHOR CONTRIBUTIONS

**Shin Takasawa:** Conceptualization (lead); data curation (equal); formal analysis (equal); investigation (equal); methodology (equal); project administration (lead); writing – original draft (lead); writing – review and editing (equal). **Mai Makino:** Investigation (equal); methodology (equal); writing – review and editing (equal). **Akiyo Yamauchi:** Investigation (equal); methodology (equal); writing – review and editing (equal). **Sumiyo Sakuramoto‐Tsuchida:** Investigation (equal); methodology (equal); writing – review and editing (equal). **Rina Hirota:** Investigation (equal); methodology (equal); writing – review and editing (equal). **Ryusei Fujii:** Investigation (equal); methodology (equal); writing – review and editing (equal). **Keito Asai:** Investigation (equal); methodology (equal); writing – review and editing (equal). **Yoshinori Takeda:** Investigation (equal); methodology (equal); writing – review and editing (equal). **Tomoko Uchiyama:** Investigation (equal); methodology (equal); writing – review and editing (equal). **Ryogo Shobatake:** Investigation (equal); methodology (equal); writing – review and editing (equal). **Hiroyo Ota:** Investigation (equal); methodology (equal); writing – review and editing (equal).

## CONFLICT OF INTEREST STATEMENT

The authors confirm that there are no conflicts of interest.

## Data Availability

The data are available on request from the authors.
